# Recent Progress in the Synthesis of 3D Complex Plasmonic Intragap Nanostructures and Their Applications in Surface-Enhanced Raman Scattering

**DOI:** 10.3390/bios14090433

**Published:** 2024-09-06

**Authors:** Li Ma, Keyi Zhou, Xinyue Wang, Jiayue Wang, Ruyu Zhao, Yifei Zhang, Fang Cheng

**Affiliations:** State Key Laboratory for Organic Electronics and Information Displays & Jiangsu Key Laboratory for Biosensors, Institute of Advanced Materials (IAM), Jiangsu National Synergetic Innovation Center for Advanced Materials (SICAM), Nanjing University of Posts and Telecommunications, Nanjing 210023, China

**Keywords:** intragap, near field, nanostructure synthesis, localized surface plasmon resonance, surface-enhanced Raman scattering, biosensing

## Abstract

Plasmonic intragap nanostructures (PINs) have garnered intensive attention in Raman-related analysis due to their exceptional ability to enhance light–matter interactions. Although diverse synthetic strategies have been employed to create these nanostructures, the emphasis has largely been on PINs with simple configurations, which often fall short in achieving effective near-field focusing. Three-dimensional (3D) complex PINs, distinguished by their intricate networks of internal gaps and voids, are emerging as superior structures for effective light trapping. These structures facilitate the generation of hot spots and hot zones that are essential for enhanced near-field focusing. Nevertheless, the synthesis techniques for these complex structures and their specific impacts on near-field focusing are not well-documented. This review discusses the recent advancements in the synthesis of 3D complex PINs and their applications in surface-enhanced Raman scattering (SERS). We begin by describing the foundational methods for fabricating simple PINs, followed by a discussion on the rational design strategies aimed at developing 3D complex PINs with superior near-field focusing capabilities. We also evaluate the SERS performance of various 3D complex PINs, emphasizing their advanced sensing capabilities. Lastly, we explore the future perspective of 3D complex PINs in SERS applications.

## 1. Introduction

Surface-Enhanced Raman Scattering (SERS) is a powerful and versatile analytical technique capable of providing chemical fingerprint spectra for various target molecules [1,2,3,4]. The principle of SERS is based on Raman scattering, where incident light interacts with molecular vibrations, resulting in inelastic scattering that provides a distinctive molecular fingerprint. Historically, the inherently weak Raman effect, with only a small fraction of incident photons being inelastically scattered, limited the sensitivity of Raman spectroscopy. This limitation was overcome in 1974 when Fleischmann and colleagues observed enhanced Raman signals from pyridine adsorbed on roughened silver electrodes [5]. Although initially attributed to increased surface area, subsequent studies by Jeanmaire [6] and Albrecht [7] clarified that the enhancements were due to electromagnetic effects. This discovery marked the inception of SERS as a potent spectroscopic technique. Nowadays, SERS has been widely used in the fields of chemical and biological sensing due to its high sensitivity and molecular specificity [8,9,10,11]. The primary mechanism behind SERS is electromagnetic enhancement [12,13,14], which occurs when plasmonic nanostructures support localized surface plasmon resonance (LSPR). LSPR is triggered when incident photon frequency aligns with the collective oscillation frequency of conducting electrons [15,16,17]. These resonances generate intense localized electromagnetic fields, particularly in regions known as “hot spots”, such as nanogaps [18,19,20] or tips of nanostructures [21,22,23]. Molecules located in these hot spots experience a dramatically enhanced local electromagnetic field, leading to significant amplification of their Raman signals. Electromagnetic enhancement can amplify Raman signals by 10^4^ to 10^11^ folds, which is significantly higher than the 10 to 10^2^-fold enhancement provided by chemical enhancement, which is the secondary SERS mechanism [24,25,26]. Due to the high sensitivity of LSPR to the morphology and composition of nanoparticles, considerable efforts have been devoted to designing and preparing plasmonic nanostructures that focus electromagnetic fields effectively [27,28,29,30].

Creating narrow gaps in plasmonic nanostructures is a promising approach to achieving superior SERS activity due to their intense hot spots and highly localized fields. There are mainly two types of nanogaps: intergaps between adjacent nanostructures and intragaps within an individual nanostructure [31]. Despite advances in using polymers [32], DNA [33], or DNA origami [34] to create plasmonic intergaps, these structures often lack sufficient rigidity, making them prone to collapse during practical applications. In contrast, plasmonic intragap nanostructures (PINs) enable the precise engineering of narrow gaps within individual nanostructures, ensuring uniform and stable electromagnetic fields. This inherent robustness makes PINs a more reliable choice for applications requiring consistent structural integrity and performance. Core–shell structures, comprising a core particle enveloped by a complete shell with a nanoscale spacer, represent the most extensively studied form of PINs [35,36,37,38,39,40,41,42]. The synthesis process involves attaching Raman dye-modified molecules, like thiolated oligonucleotides, to the metal core prior to shell formation. This method ensures the creation of uniform nanogaps between the core and the shell, yielding highly reproducible SERS signals. However, the enclosing shell may limit analyte access to the hot spots, thus potentially hindering label-free SERS sensing. Moreover, the presence of Raman dyes may alter the morphology of nanogap, introducing variability in SERS signals.

Recently developed three-dimensional (3D) complex PINs have significantly advanced the capabilities of SERS by enhancing both sensitivity and reproducibility [43]. Unlike core–shell PINs, these 3D complex PINs incorporate open intragaps that circumvent the limitations posed by enclosed structures. Additionally, the 3D architectures not only offer more space for hot-spot engineering but also provide polarization-independent SERS activity. However, research on 3D complex PINs still encounters several challenges. Firstly, the controlled preparation of highly uniform 3D complex PINs is essential for developing SERS applications. Although wet-chemical synthesis is more cost-effective than top-down lithographic methods [44,45,46,47], it demands careful design of synthetic pathways to fabricate complex nanostructures. Secondly, understanding the relationship between structure and properties is crucial for designing 3D complex PINs that exhibit superior SERS activity. Traditional ensemble measurements of SERS spectra, which often suffer from averaging effects, are inadequate for accurately delineating this relationship. In contrast, single-particle SERS measurements provide a detailed analysis of individual nanostructures, offering insights into their specific enhancement capabilities [48]. This approach not only identifies factors influencing the SERS signal but also assists in optimizing nanostructures for enhanced SERS performance.

While recent reviews have explored the role of porous metals [49,50] and non-noble-metal materials [51,52,53] for SERS applications, there remains a lack of comprehensive coverage of the synthesis strategies for 3D complex PINs and their applications in SERS. This review aims to fill this gap by focusing on 3D complex PINs, with a particular emphasis on gold-based nanoparticles. We first summarize the representative synthesis pathways for 3D complex PINs using wet-chemical methods. The discussion then moves to two primary strategies for structural tuning: outer frame engineering and inner structure engineering, both starting from basic 3D nanoframes. Drawing on insights from the single-particle SERS analysis, we then discuss how structural parameters can be fine-tuned to optimize near-field concentration. We also investigate the applications of these structures in SERS sensing. Lastly, we address the current challenges and outline potential future research directions in this field.

## 2. Synthesis of 3D Complex PINs

Producing highly uniform 3D complex PINs is crucial for achieving reliable SERS signals due to the technique’s sensitivity to the size, shape, and composition of nanoparticles. However, achieving precise, scalable, and reproducible synthesis remains a significant challenge. While wet-chemical synthesis can produce large quantities of particles efficiently, it demands an in-depth understanding of reaction mechanisms involving multiple reactants, complexes, and surfactants. This complexity provides opportunities for extensive optimization of the particle structure to enhance SERS activity.

**Figure 1:**
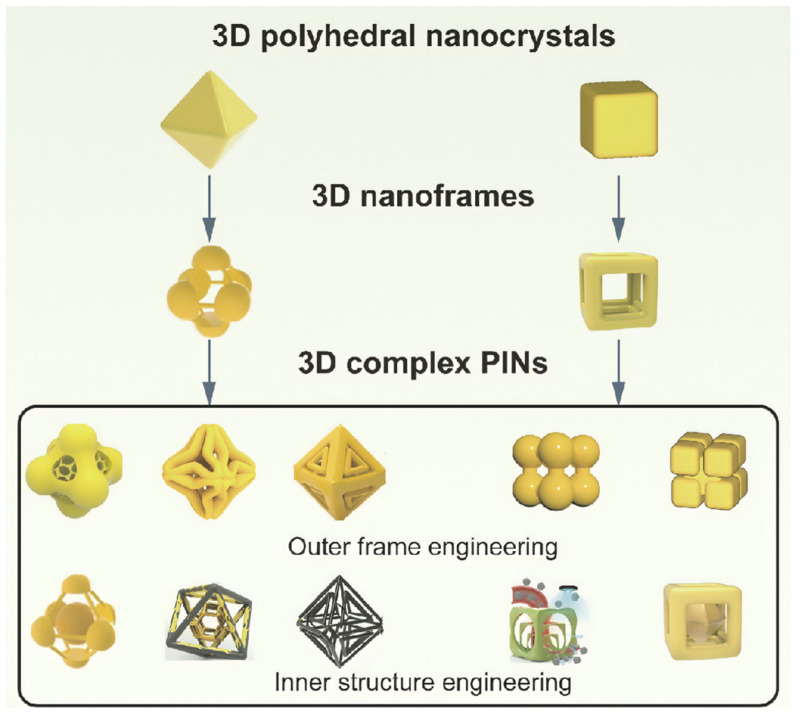
Schematic illustration of synthetic strategies for 3D complex PINs from polyhedral nanocrystals.

Three-dimensional polyhedral nanocrystals, such as octahedrons and cubes, possess distinct crystallographic facets that can be harnessed to create areas of high electromagnetic activity. These structures are pivotal for the formation of 3D complex PINs. In this section, we review the synthetic strategies of 3D complex PINs in the past 5 years, specifically focusing on those with octahedral and cubic configurations. We begin by describing the synthesis of simple nanoframes from octahedral and cubic nanocrystals and then proceed to summarize how these simple frames are further engineered—both outer frame and inner structure—to create 3D complex PINs (Figure 1).

### 2.1. Three-Dimensional Nanoframes Transformed from Polyhedral Nanocrystals

**Figure 2:**
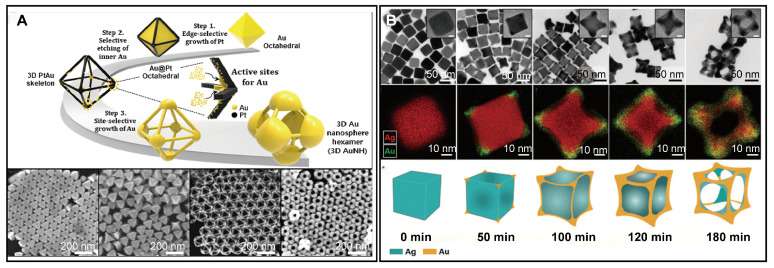
Structural transformation from 3D polyhedral nanocrystals to 3D nanoframes. (**A**) Synthesis of 3D Au nanosphere hexamer by a three-step method, with the corresponding products in each step. Copyright 2020 American Chemical Society [43]. (**B**) Structural evolution from Ag nanocubes to AuAg cubic nanoframes. Copyright 2022 American Chemical Society [54].

Three-dimensional nanoframes are particularly valuable for SERS sensing due to their open structure and abundant intragaps that enhance near-field focusing and analyte access. Unlike the self-assembly strategies that connect nanoparticles with linker molecules, solution-phase synthesis typically starts with 3D polyhedral nanocrystals. In 2020, Yoo and coworkers devised a multistep synthetic strategy to prepare a 3D Au nanosphere hexamer composed of six Au nanospheres linked by thin metal bridges [43]. This strategy is divided into three steps: (1) edge-selective growth of Pt, (2) selective etching of inner Au, and (3) site-selective growth of Au (Figure 2A). The synthesis begins with highly homogenous Au octahedral templates. For edge-selective growth of Pt, Ag is initially deposited on the templates, followed by a galvanic replacement reaction with Pt^4+^ ions. This reaction results in the selective deposition of Pt on the edges of the Au templates due to a significant lattice constant mismatch between Au and Pt. Selective etching of inner Au is accomplished through a comproportionation reaction between HAuCl_4_ and inner Au atoms, forming a 3D PtAu skeleton. The final step involves the site-selective growth of Au, which is facilitated by reducing HAuCl_4_ with ascorbic acid (AA). This critical step determines the morphology of the final 3D Au nanosphere hexamer. Control experiments indicate that selective growth optimally occurs under low pH without adding AgNO_3_, while at high pH, the Au reduction rate increases, facilitating more uniform growth. The significant lattice constant mismatch between Au and Pt enhances the site-selective growth, while the introduction of Ag layers mediates this mismatch, leading to the homogeneous growth of Au on the PtAu skeleton. By finely tuning these growth conditions, the 3D Au nanosphere hexamer with various geometries can be synthesized, which is instrumental for systematically studying the relationship between structure and SERS activity.

Despite prior achievements in creating 3D octahedral nanoframes, the multi-step strategy requires precise control at every stage. Consequently, there is a demand for simpler synthetic methods. Our group recently developed a one-step galvanic replacement method to synthesize AuAg cubic nanoframes with large openings on each facet [54]. Traditional galvanic replacement reactions between Ag and Au^3+^ ions typically form hollow nanostructures with a porous network on each facet [55,56,57,58]. To fabricate nanoframes with substantial openings, additional etching is usually necessary. Although gold precursors can act as etchants, their interaction with Ag dealloying complicates the control over shell thickness and porosity. Other etchants like Fe(NO_3_)_3_, NH_4_OH, or H_2_O_2_ have been explored, but controlling the final morphology remains challenging. Using Na_3_(AuS_2_O_3_), a univalent gold precursor, we observe a continuous concaving and hollowing-out process, distinct from traditional galvanic replacement (Figure 2B). Control experiments showed that the reaction rate, capping agent, and Na_2_S_2_O_3_ are crucial for shaping the morphology of the final product. This facile method successfully transforms Ag nanocubes into AuAg cubic nanoframes. By simply altering the capping agent from hexadecyltrimethylammonium bromide (CTAB) to polyvinylpyrrolidone (PVP), the product changes from nanoframes to nanoboxes without apparent holes on each facet. Similarly, this approach can efficiently transform Ag triangular plates and Ag nanowires into corresponding nanoframes. Notably, wire-like nanoframes with intragap exhibit higher SERS activity compared to enclosed nanocubes.

### 2.2. Three-Dimensional Complex PINs Constructed by Outer Frame Engineering of 3D Nanoframes

**Figure 3:**
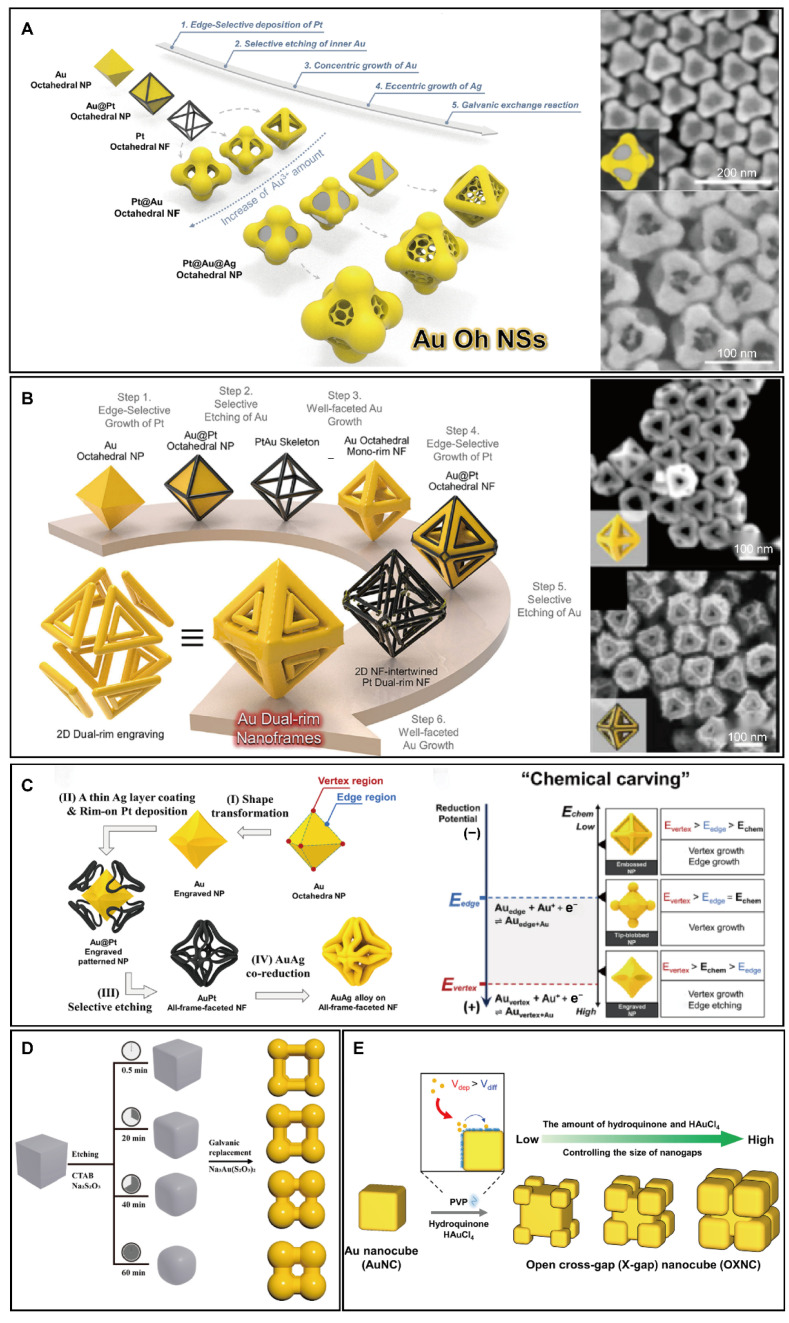
Rational design of synthesis for 3D complex PINs by outer frame engineering of 3D nanoframes. (**A**) Au octahedral nanosponges. Copyright 2023 American Chemical Society [59]. (**B**) Au dual frame-engraved nanoframes. Copyright 2022 Springer Nature [60]. (**C**) AuAg all-frame-faceted tripod nanoframes. Copyright 2022 American Chemical Society [61]. (**D**) AuAg nanosphere octamer. Copyright 2024 American Chemical Society [62]. (**E**) Au cross-gap nanocubes. Copyright 2024 American Chemical Society [63].

Despite their potential for near-field focusing, 3D nanosphere hexamers and AuAg cubic nanoframes still face challenges in effectively confining the electromagnetic field. In the past 5 years, advancements in structural design have further enhanced near-field focusing. The open spaces on each facet of 3D nanoframes offer opportunities to engineer additional hot spots for improved near-field confinement. Following previous research, Kwon developed a five-step synthesis method for Au octahedral nanosponges, which incorporate porous structure on each facet, significantly increasing the number of hot spots [59]. The synthesis begins with Au octahedral templates, with the first three steps mirroring those used in the synthesis of 3D-connected hexamers. After producing Pt@Au octahedral nanoframes from the third step, eccentric Ag growth (Step 4) and a galvanic replacement reaction (Step 5) create the 3D octahedral nanosponges (Figure 3A). In Step 4, Ag is selectively grown at the inner domains of the Pt@Au octahedral nanoframes using CTAB as a surfactant, taking advantage of the higher surface energy of the inner domains. The sequential galvanic replacement reaction between the inner Ag and HAuCl_4_ leads to the formation of porous structures on the facets of Pt@Au octahedral nanoframes.

While Au octahedral nanosponges offer numerous hot spots for effective near-field focusing, their randomly distributed hot spots complicate the reproducibility of SERS signals. To improve facet control, Hilal developed a six-step synthesis method for 3D dual-frame-engraved nanoframes (Figure 3B) [60]. These complex structures feature two-dimensional (2D) dual rims on each facet. Initially, a 3D PtAu skeleton is formed from Au octahedral templates using a two-step process. The synthesis then involves well-faceted Au growth and a second edge-selective growth of Pt, followed by a second selective etching of Au and another well-faceted Au growth. Unlike previous methods that targeted selective Au growth at the vertices of the 3D PtAu skeleton, the conditions here are adjusted to promote uniform Au growth along the Pt edges of the PtAu skeleton, resulting in Au octahedral mono-rim nanoframes with triangular facets. These structures are pivotal for creating dual-frame-engraved 3D nanoframes. The sharp edges of the Au octahedral mono-rim nanoframes facilitate the growth of Pt on both the inner and outer edges, leading to the formation of Au@Pt dual-rim octahedral nanoframes. Subsequent selective etching and well-faceted growth of Au transform these nanoframes into Au dual-rim octahedral nanoframes. The resulting dual rims on each facet of these octahedral frames create narrow intragaps, significantly enhancing the electromagnetic-focusing capability.

Another approach to outer frame engineering involves synthesizing AuAg all-frame-faceted nanoframes with a multi-tripod design on each facet. This four-step synthesis process includes: (1) shape transformation, (2) selective growth of Pt, (3) selective etching of Au, and (4) AuAg co-reduction (Figure 3C) [61]. The cornerstone of this strategy is selective chemical carving, which successfully transforms Au octahedral templates into intricately engraved nanoparticles with 24 edges. This transformation is achieved by precisely tuning the electrochemical potential of the solution (*E*_chem_) by adjusting the concentration of CTAB and AA. As illustrated in Figure 3C, the standard redox potential at the vertex (*E*_vertex_) exceeds that at the edges (*E*_edge_). By setting the electrochemical potential of the solution between the redox potential of vertex and edges (*E*_vertex_ > *E*_chem_ > *E*_edge_), growth is promoted at the vertex while etching predominates at the edges, leading to the creation of intricately engraved nanoparticles. Following selective etching of Au and co-reduction of AuAg, all-frame-faceted nanoframes are produced. This complex structure, rich in intragaps, demonstrates significant potential for efficient near-field confinement.

To engineer AuAg cubic nanoframes for enhanced near-field concentration, our group developed a facile one-pot method to synthesize 3D nanosphere octamer [62]. This method involves a selective etching step using Na_2_S_2_O_3_ to round the corners of Ag nanocubes (Figure 3D). Following this, without isolating the Ag nanocubes from the solution, a galvanic replacement reaction is used to create 3D AuAg nanosphere octamer. This one-pot approach without isolation of intermediates significantly simplifies the synthesis process. The etching duration is pivotal as adjusting it allows for the creation of Ag nanocubes with varying radii of curvature at the corners, thus enabling precise control over the geometry of the final 3D nanosphere octamers. These 3D nanosphere octamers, with varied structural parameters, serve as a basis for exploring the relationship between structure and SERS activity. Moreover, the Jwa-Min Nam group introduced a site-specific growth method that enables the selective growth of budding Au structures at the corners of cubic Au templates, producing cross-gap Au nanocubes (Figure 3E) [63]. Maintaining a deposition rate higher than the diffusion rate of Au (V_dep_ > V_diff_) is crucial for the synthesis. Adjusting the concentration of the reducing agent (hydroquinone), the stabilizer (PVP), and the sharpness of the corners of the Au templates influences this site-specific growth. By finely tuning the amounts of the reducing agent and Au precursor, cross-gap Au nanocubes with intragaps ranging from 3 to 20 nm were successfully synthesized, significantly benefiting SERS sensing due to their open intragap structure.

### 2.3. Three-Dimensional Complex PINs Constructed by Inner Structure Engineering of 3D Nanoframes

**Figure 4:**
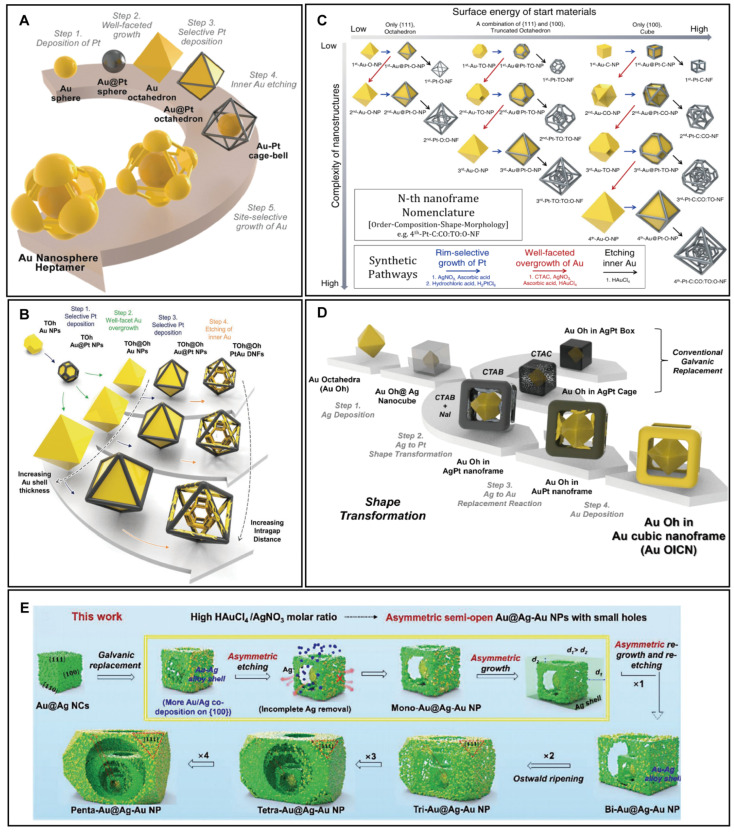
Rational design of synthesis for 3D complex PINs by inner structure engineering of 3D nanoframes. (**A**) Au nanosphere heptamer. Copyright 2024 American Chemical Society [64]. (**B**) Truncated-octahedral@octahedral PtAu dual nanoframes. Copyright 2023 Wiley-VCH GmbH [65]. (**C**) Multi-layered Au nanoframes. Copyright 2022 Springer Nature [66]. (**D**) Au octahedra@Au cubic nanoframes. Copyright 2024 American Chemical Society [67]. (**E**) Multi-layered AuAg nanoframes. Copyright 2023 Wiley-VCH GmbH [68].

In addition to the open spaces on nanoframes’ facets, the substantial internal space can also be engineered to further confine the electromagnetic near field. A strategy involves inserting an Au@Pt nanosphere inside an octahedral nanoframe, effectively reducing intragap size [64]. This synthesis closely resembles that used for the 3D Au nanosphere hexamer, with the main difference being the substitution of Au octahedral templates with Au spheres (Figure 4A). A critical step involves coating the Au sphere with a 7 nm-thick layer of Pt, which uniformly covers the sphere and protects it from etching. This protective layer enables the formation of an AuPt cage bell structure after selective etching of the inner Au. Subsequent site-selective growth of Au completes the structure, resulting in an Au nanosphere heptamer. This design, featuring an additional Au nanosphere within the nanoframe, creates more hot spots between nanospheres and nanogaps, significantly enhancing the electromagnetic near field.

Furthermore, the Park group has explored more intricate designs by nesting nanoframes within nanoframes [65]. Starting with truncated-octahedral Au nanoparticles, site-selective deposition of Pt, exclusively on the edges, leads to the formation of truncated-octahedral Au@Pt nanoparticles (Figure 4B). This protective thin layer of Pt shields the underlying Au from etching, which is crucial for the synthesis. The process involves a sequential overgrowth of Au, selective deposition of Pt, and etching of inner Au, resulting in truncated-octahedral@octahedral PtAu dual nanoframes. By repeating these synthetic steps, Yoo and colleagues successfully produced multi-layered nanoframes (Figure 4C) [66]. Different starting templates were employed to achieve varied morphologies in the third and fourth nanoframes, demonstrating precise control over each synthesis step and underscoring the potential of these complex structures for advanced applications.

For cubic nanoframes, Oh and colleagues embedded an Au octahedra inside a cubic nanoframe [67]. The narrow intragaps formed between the sharp tips of the inner octahedra and the edges of the outer cubic nanoframe effectively confine the electromagnetic near field. The synthesis involves the following steps: (1) growth of Ag on Au octahedra to form Au octahedra@Ag nanocubes, (2) galvanic replacement between Ag and Pt^2+^ to form Pt nanoframes, (3) galvanic replacement between Ag and Au^3+^ to form Au octahedra in AuPt nanoframe, and (4) regrowth of Au (Figure 4D). The critical step of the synthesis is the structural transformation from Au octahedra@Ag nanocubes to Au octahedra@AgPt nanoframes. By using CTAB and NaI, the replacement of Ag and Pt^2+^ preferential occurs on the flat (100) facets, producing the Au octahedra@AgPt nanoframes. The subsequent steps successfully transform the outer AgPt nanoframes to Au nanoframes, demonstrating precise control over the final product’s structure.

In a complex multi-layered architecture, a nanoframe was integrated within a cubic nanoframe [68]. Starting from Au nanosphere cores, Au@Ag nanocubes are produced after Ag overgrowth on Au seeds (Figure 4E). Through a series of galvanic replacement reactions and etching with H_2_O_2_, AuAg nanoframes are formed. Repeating the galvanic replacement and Ag overgrowth steps allows for the creation of multi-layered AuAg nanoframes. To achieve asymmetric multi-layered nanoframes, the molar ratio of HAuCl_4_ to AgNO_3_ was adjusted to 0.16, facilitating asymmetric Ag etching. By varying the duration of galvanic replacement, the number of layers in the multi-layered nanoframes could be controlled, ranging from one to five layers.

## 3. Single-Particle SERS Analysis of 3D Complex PINs

**Figure 5:**
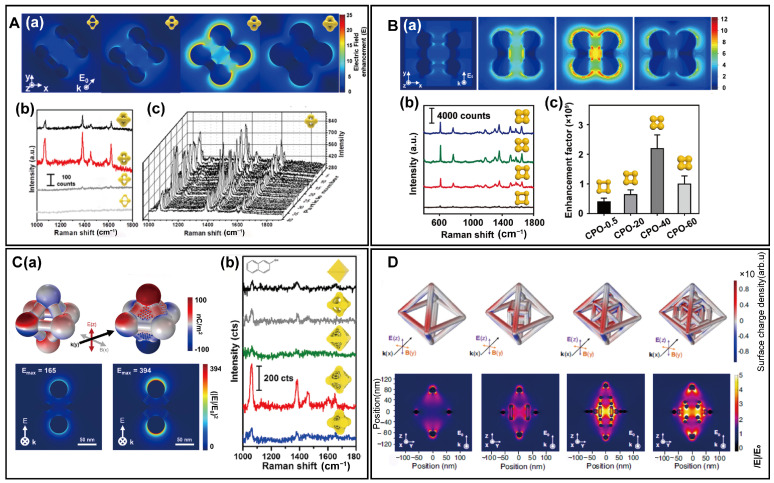
Single-particle SERS activity of 3D complex PINs. (**A**) Au nanosphere hexamer. (**a**) FEM calculations of near-field distribution. (**b**) Single-particle SERS measurements of Au nanosphere hexamer with different structures. (**c**) Reproducibility of single-particle SERS measurements. Copyright 2020 American Chemical Society [43]. (**B**) Au nanosphere octamer. (**a**) Calculated near-field distributions. (**b**) Single-particle SERS measurements of Au nanosphere octamer with different structures. (**c**) Calculated enhancement factors. Copyright 2024 American Chemical Society [62]. (**C**) Au octahedral nanosponges. (**a**) Calculated near-field distributions. (**b**) Single-particle SERS measurements of Au octahedral nanosponges with different structures. Copyright 2023 American Chemical Society [59]. (**D**) Calculated near-field distributions of multi-layered nanoframes with different structures. Copyright 2022 Springer Nature [66].

In contrast to plasmonic intergap nanostructures where particles are separated by nanogaps, 3D complex PINs typically consist of nanoparticles interconnected by metal bridges. This unique configuration significantly impacts the distribution of the near field. To investigate the relationship between structure and near-field focusing capabilities, single-particle SERS measurements were conducted on a variety of 3D complex PINs. By fine-tuning the synthetic conditions, 3D Au nanosphere hexamers varying in nanosphere size and bridge thickness were successfully prepared [43]. Finite-element method (FEM) calculations indicate that the electromagnetic near field is primarily concentrated at vertices, bridges, and intragap regions of the nanoparticles (Figure 5A(a)). This pattern of distribution starkly contrasts with that observed in assembled nanoparticles lacking metal bridges, highlighting the critical role of metal bridges in near-field focusing. Intriguingly, the maximum near field is not observed in nanoparticles with the narrowest intragaps but, rather, in those with the most significant disparities between vertices and bridge thickness, revealing a previously unidentified characteristic of 3D complex PINs with metal bridges. Additionally, the single-particle SERS measurements demonstrated the highest intensity on the same nanoparticle, aligning with the FEM results (Figure 5A(b,c)).

Similar results were also observed in serials of 3D AuAg nanosphere octamers, where the most intense near-field focusing and Raman intensity were noted in nanoparticles with moderate intragaps—not too large or too small [62] (Figure 5B(a,b)). The highest enhancement factor recorded in this series reaches up to 2.2 × 10^9^ (Figure 5B(c)). For Au octahedral nanosponges, the porous network significantly enhanced near-field focusing. The electromagnetic enhancement value rose from 165 for Au octahedral nanoframes to 394 for Au octahedral nanosponges (Figure 5C(a)). Single-particle SERS measurements further validate the higher intensities in Au octahedral nanosponges compared to nanoframes. Moreover, there exists a trade-off between the size of the vertices and the inner sponge domain. An increase in vertex size leads to a decrease in the size of the inner sponge domain. If the vertex size becomes too large, it compresses the sponge domain, diminishing near-field focusing. Conversely, if the vertex size is too small, it fails to activate near-field focusing effectively. Therefore, the highest single-particle SERS intensities are found in nanoparticles with optimized structural parameters. Moreover, for multi-layered nanoframes, frequency-domain time-difference (FDTD) calculations indicate that electromagnetic enhancement intensifies with each added layer, highlighting the potential of layered structures to improve SERS performance (Figure 5D).

## 4. Application of 3D Complex PINs in SERS Detection

**Figure 6:**
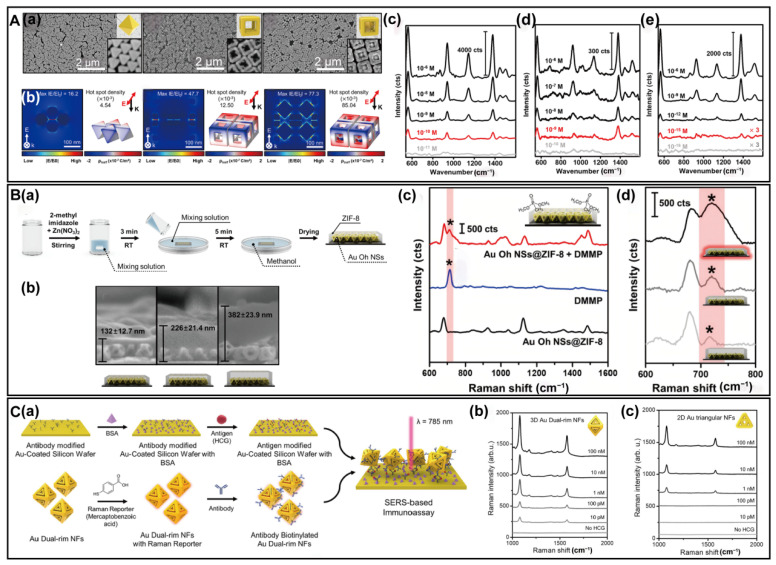
Potential SERS applications using 3D complex PINs. (**A**) SERS detection using self-assembled 3D complex PINs. (**a**) SEM images of self-assembled monolayer of Au octahedral, Au cubic nanoframes, and Au octahedral@Au cubic nanoframes. (**b**) Calculated near-field distributions of Au octahedral, Au cubic nanoframes, and Au octahedral@Au cubic nanoframes. (**c**–**e**) Bulk SERS spectra from Au octahedral (**c**), Au cubic nanoframes (**d**), and Au octahedral@Au cubic nanoframes (**e**). Copyright 2024 American Chemical Society [67]. (**B**) SERS detections of gas analytes using 3D complex PINs. (**a**) Schematic illustration for synthesis of an Au octahedral nanosponges@ZIF-8 film substrate. (**b**) Cross-section SEM images of SERS substrates with different thicknesses. (**c**) SERS spectra using different substrates. (**d**) SERS spectra using Au octahedral nanosponges@ZIF-8 substrate with different thicknesses. The characteristic peak of DMMP at 719 cm^−1^ is marked as asterisk. Copyright 2023 American Chemical Society [59]. (**C**) SERS immunoassay of HCG using 3D complex PINs. (**a**) Schematic illustration of SERS immunoassay of Au dual-rim nanoframes. (**b**,**c**) SERS spectra using 3D Au dual-rim nanoframes (**b**) and 2D Au triangular nanoframes (**c**) as probes. Copyright 2022 Springer Nature [60].

Thanks to their strong near-field focusing capabilities as demonstrated by single-particle SERS, 3D complex PINs have been advanced to develop bulk SERS substrates for chemical and biological sensing applications. Unlike single-particle SERS substrates, which feature intragaps, bulk SERS substrates combine intragaps within individual particles and intergaps among multiple particles. Due to the high uniformity of the 3D complex PINs, large areas of 2D monolayer films were formed by self-assembly at water–hexane interfaces. For comparison, bulk SERS substrates were built up from Au octahedral, Au cubic nanoframes, and Au octahedral@Au cubic nanoframes, respectively [60]. These close-packed monolayers possess a substantial number of hot spots through inter- and intra-coupling among the nanoparticles (Figure 6A(a)). The FDTD calculations for the assembled nanoparticles reveal the highest electromagnetic enhancement and hot-spot density for Au octahedral@Au cubic nanoframe assembly (Figure 6A(b)). SERS detection of thiram utilizing these substrates reveals detection limits of 10^−10^ M for Au octahedral, 10^−9^ M for Au cubic nanoframes, and 10^−15^ M for Au octahedral@Au cubic nanoframes, respectively (Figure 6A(c–e)). The exceptionally high sensitivity was attributed to the synergistic effects of the inner Au octahedral and outer Au cubic nanoframes, which produce significant electromagnetic enhancement and large amounts of hot spots.

In addition to solution-based SERS detection, 3D complex PINs were employed to detect analytes in the gas phase. Au octahedral nanosponges, with their large surface area and abundant built-in hot spots, achieved a detection limit as low as 10 ppb for weakly adsorbing analytes such as 2-chloroethyl phenyl sulfide [54]. The excellent gas capture capability of Metal-Organic Frameworks (MOFs) proved advantageous for SERS sensing. SERS substrates incorporating 3D complex PINs were thus coated with MOF films, created by depositing a solution of 2-methylimidazole and zinc nitrate hexahydrate onto a monolayer film of Au octahedral nanosponges (Figure 6B(a)). By adjusting the reaction time, hybrid SERS substrates with different MOF thicknesses were obtained (Figure 6B(b)). Utilizing nonadsorbing dimethyl methyl phosphonate (DMMP) as a model analyte, these hybrid SERS substrates successfully detect DMMP down to 10 ppm (Figure 6B(c)), with thinner MOF layers resulting in higher SERS intensity (Figure 6B(d)).

Furthermore, 3D complex PINs have been applied in SERS immunoassays for human chorionic gonadotropin (HCG) [66]. The Au dual-rim nanoframes are first labeled with a Raman reporter and then modified with HCG antibodies (Figure 6C(a)). Concurrently, the substrates are modified with antigens. Upon mixing the antigen-modified substrates with antibody-biotinylated Au dual-rim nanoframes, the specific antigen–antibody interaction between antigen and antibody brings the Au dual-rim nanoframes with Raman reporter close to the substrate, facilitating the detection of HCG. For comparison, both 3D Au dual-rim nanoframes and 2D Au triangular nanoframes were used to label the Raman reporter. Significantly higher Raman intensity and a much lower detection limit are observed for 3D Au dual-rim nanoframes compared to 2D Au triangular nanoframes, demonstrating their superior SERS-detection capabilities (Figure 6C(b)).

## 5. Conclusions and Outlook

In the past years, the rational synthesis and SERS applications of 3D complex PINs have attracted significant attention. This review highlights the latest advancements in the design and synthesis of 3D complex PINs via wet chemistry and their utilization in SERS sensing. Initially, simple 3D nanoframes are crafted from polyhedral nanocrystals. To develop more intricate structures, two main strategies—outer frame and inner structure engineering—are employed, facilitating enhanced near-field focusing. Through carefully designed synthetic pathways, 3D complex PINs with high uniformity in shape and size are manufactured. These nanostructures can be used to prepare SERS substrates for both the solution-phase and gas-phase detection.

Despite the current progress, several challenges remain. Firstly, achieving precise, scalable, and reproducible synthesis of 3D complex PINs in a simple and facile way remains difficult. Among the myriad studies, only Au nanosphere octamer and Au cross-gap nanocubes have been synthesized in one or two steps, whereas most other structures require more complex, multi-step synthetic pathways. Such multi-step strategies necessitate exacting control at each phase, complicating the synthesis process. Secondly, the relationship between structure and near-field focusing on 3D complex PINs demands in-depth study. Due to their complex architecture and interconnected metal bridges, these nanostructures exhibit complicated near-field distributions, requiring detailed, case-specific research. Thirdly, there is a limited number of studies employing 3D complex PINs in SERS detection, which restricts our understanding of their practical applications. Despite these challenges, 3D complex PINs show immense promise for SERS sensing. With the ongoing development of new synthetic strategies, it is anticipated that these nanostructures will find increasing applications in the field of SERS sensing.

## Data Availability

Not applicable.
